# Supplementary material for: EmbedTAD Using Graph Embedding and Unsupervised Learning to Identify TADs from High-Resolution Hi-C Data
Source: Commun Biol. 2025 Dec 9;9:7. doi: 10.1038/s42003-025-09224-z (PMC12764586; doi:10.1038/s42003-025-09224-z)
Supplement: Supplementary file 2 — Supplementary Material [file 42003_2025_9224_MOESM2_ESM.pdf]

EmbedTAD Using Graph Embedding and Unsupervised Learning to Identify TADs from High-Resolution Hi-C Data

H. M. A. Mohit Chowdhury[1,2] and Oluwatosin Oluwadare[1,2*]

[1]Department of Computer Science and Engineering, University of North Texas, 1155 Union Circle, Denton, 76203, TX, USA.
[2]Center for Computational Life Sciences, University of North Texas, Denton, 76207, TX, USA.

*Corresponding author(s). E-mail(s): oluwatosin.oluwadare@unt.edu;
Contributing authors: h.m.a.mohitchowdhury@my.unt.edu;

Supplementary Information

Hyperparameter Search

EmbedTAD converts the Hi-C contact matrix into graph data and uses embedding to represent this graph in lower dimensions, making it computationally efficient. Suppose we have a $n \times m$ matrix, where m represents the features and n represents the samples. After applying the embedding algorithm, the new shape of the data will be $n \times e$, where e denotes the number of embedded features in the lower-dimensional space and $e < n$. Determining the optimal embedded feature size (e) is the most challenging step to achieve optimal result. Since the Hi-C contact matrix varies in size across different chromosomes and organisms, selecting an appropriate embedding size for these variations is a crucial step in EmbedTAD's architecture to preserve all essential features. To determine an optimal embedding size for our problem, we ran EmbedTAD with different embedding sizes using simulated data from Forcato et al. [1]. We used their data at varying noise levels (4, 8, 12, 16, 20) at 40Kb resolution, as different noise levels help identify the optimal embedding size for various chromosomes and organisms.

Performance Comparison of CPU and GPU Implementations of EmbedTAD

We developed both CPU and GPU implementations of EmbedTAD to provide easy access for users. The GPU implementation is faster than the CPU version, significantly reducing both running time and memory usage. Graph creation, which represents the relationships among different nodes, is crucial but also time and memory-intensive. While the CPU and GPU implementations use different libraries for graph creation, the embedding and clustering processes remain the same. To validate the CPU and GPU implementations, we conducted computational analyses using simulated data across all noise levels. We observed minimal differences in MoC scores between the CPU and GPU versions (Supplementary Figure 19C), where GPU versions shows better MoC in most cases. Additionally, we compared TAD quality and found that the score for both CPU and GPU versions followed similar trends at 4, 8, 12, and 16 noise levels, and GPU version showed better TAD quality in all noise level compared to CPU version. It is worth noting that at higher noise levels, the GPU version showed a consistently rising TAD quality across all cases (Supplementary Figure 19C). This analysis demonstrates considerable consistency between the CPU and GPU versions of EmbedTAD, providing a comprehensive comparison between the two. We also recorded graph creation and embedding times, and the GPU version achieved 9× faster graph creation time and 2× faster embedding time compared to the CPU version.

Determining Optimal Embedding Size

First, we performed Principal Component Analysis (PCA) to determine a probable range for the embedded size on simulated Hi-C data and got embedding sizes ranging from 385 to 480. To verify this prediction, we extensively ran EmbedTAD with embedding sizes ranging from 20 to 520. We used the Measure of Concordance (MoC) [2] as our primary metric for selecting the optimal embedding size. The MoC score, where 0 denotes the lowest MoC and 100 the highest, is used to compare the output from EmbedTAD with the real TADs for the synthetic data. Therefore, high MoC values imply EmbedTAD's strong confidence in concordance with the true synthetic TADs, whereas lower values indicate a lesser concordance. We found that an embedding size of 455 produced the highest MoC across all noise levels (Supplementary Figure 19A). Additionally, as shown in Supplementary Figure 19A, embedding sizes ranging from 300 to 500 demonstrated higher MoC, which corroborates the PCA prediction. We observed that MoC values became steady around an embedding size of 455 across different noise levels (Supplementary Data 1). Based on these findings, we selected an embedding size of 455 as the optimal value, as it consistently produced the highest MoC across all noise levels.

Validation of Optimal Embedding Size Using TAD Quality Metric

Primarily, we used MoC to determine the embedding size, and we found that an embedding size of 455 provides an optimal result across all noise levels (Section 2). However, it is essential to verify the selected embedding size with other metrics to ensure its robustness. Importantly, a blind or unsupervised metric that does not rely

on the true TADs to determine the correctness of detected TADs is needed, so it can be applied to real Hi-C datasets for TAD identification. To do this, we chose the TAD Quality metric by Oluwadare et al. [3]. This metric works by maximizing intra-TAD interactions while minimizing inter-cluster relations in its evaluation, ensuring that TADs are well-defined. We used the TAD Quality metric and observed that a higher MoC score is correlated with a higher TAD quality at the specified embedding size. TAD quality scores were higher for embedding sizes ranging from 350 to 500 in all noise levels (Supplementary Figure 19B and Supplementary Data 2). We achieved high TAD quality scores at 4, 8, and 12 noise levels, and the overall TAD quality curve confirmed that the optimal embedding size is 455. These observations further support the selected embedding size of 455 as maintaining an optimal MoC (Supplementary Data 3).

Performance Comparison using AMI and ARI

Since TAD borders are enriched with numerous biological landmarks, including CTCF and histone markers, TAD detection techniques are typically assessed using biological studies to validate their effectiveness [4]. The lack of ground truth for actual datasets to assess a TAD caller's performance is another factor. We used Forcato et al.'s [1] synthetic dataset, which enabled us to assess the effectiveness of our approach using classical analysis. We evaluated our approach using Adjusted Mutual Information (AMI) and Adjusted Random Index (ARI) analysis, based on their usage in previous TAD detection methods [5, 6]. Predicted bins may vary but generally overlap with the actual ground truth in terms of synthetic data, and TAD regions identified by TAD callers are neither entirely similar nor exact in terms of bins. We select AMI and ARI to assess clustering quality in light of this pattern. AMI, which has a value between 0 (random match) and 1 (perfect match), calculates the cluster similarity while taking into account the random assignment of clusters. ARI, a different metric that modifies assignment randomness and ranges from -1 (worst) to 1 (perfect), compares the bins to determine whether or not they are in the same cluster. Since there may be numerous biological landmarks, these metrics measure the TAD boundary and domain assignment accuracy, which are essential for TAD identification problem and require additional biological validation. EmbedTAD showed competitive performance over other state-of-the-art TAD callers in terms of AMI and ARI scores (Supplementary Figure 3, Table S2). We found that EmbedTAD showed nearly the same mean and median values across various noise levels in this conventional assessment metric, demonstrating that our approach generates highly symmetrical values for the same synthetic Hi-C data across various noise levels. Furthermore, we found no outliers in this metrics test, demonstrating reproducible, scalable, and consistent outcomes across a range of noise levels. These findings also demonstrate that our network embedding and graph data format regularly lower the noise sensitivity. In comparison with traditional state-of-the-art techniques, this observation indicates that our approach is competitive, robust and consistent across a range of noise levels and attained a novel position by introducing new technology in the TAD detection problem.

Supplementary Table 1 TAD callers category according to their algorithm. Categorized 8 different TAD callers into 4 categories and compared them with EmbedTAD.

#	**Category**	**TAD callers**
A	Clustering	i. EmbedTAD<br>ii. ClusterTAD<br>iii. IC-Finder<br>iv. CASPIAN
B	Linear	i. TopDom<br>ii. Armatus
C	Statistical	i. HiCseg
D	Network Feature	i. Spectral

Supplementary Table 2 AMI and ARI scores using synthetic Hi-C data. Min, mean, median, and max values of AMI and ARI scores, where EmbedTAD showed competitive results without any outliers and nearly identical mean and median values.

AMI				
Algorithm	Min	Mean	Median	Max
Armatus	0.969998668	0.981896467	0.982218487	0.988787808
Caspian	0.747592396	0.868172879	0.883509127	0.932162521
ClusterTAD	0.772843868	0.812828406	0.811367587	0.854346679
EmbedTAD	0.900536621	0.910965023	0.910481888	0.921687682
HiCSeg	0.753674818	0.886086799	0.901944653	0.965147032
IC-Finder	0.764436932	0.833936021	0.84649006	0.86612884
Spectral	0.861384586	0.876066615	0.875416135	0.889114115
TopDom	0.959487214	0.970948198	0.970439816	0.987151912
ARI				
Algorithm	Min	Mean	Median	Max
Armatus	0.920425007	0.959321786	0.959641924	0.98590165
Caspian	0.180508127	0.556910953	0.60205588	0.801420875
ClusterTAD	0.241049787	0.38545023	0.38899107	0.496041101
EmbedTAD	0.656675832	0.706174672	0.704519724	0.757862566
HiCSeg	0.39822954	0.673875186	0.696360851	0.882196289
IC-Finder	0.211452146	0.504200175	0.56672881	0.628566333
Spectral	0.582321521	0.617654169	0.617416685	0.652944626
TopDom	0.893884355	0.922603355	0.922048938	0.965793155

Supplementary Table 3 Comparison of $TADadjR^2$ score on GM12878 chromosome 19 and CH12LX chromosome 18 at 5Kb and 10Kb resolution. EmbedTAD achieved a competitive score across different organisms and resolutions.

Organism	Algorithm	5Kb			10Kb		
		Min	Median	Max	Min	Median	Max
GM12878	EmbedTAD	0.55	0.62	0.86	0.52	0.68	0.91
	ClusterTAD	0.53	0.60	0.84	0.53	0.65	0.88
	IC-Finder	0.52	0.59	0.90	0.51	0.63	0.93
	CASPIAN	0.52	0.59	0.79	0.51	0.62	0.81
	TopDom	0.53	0.60	0.89	0.51	0.63	0.92
	Armatus	0.54	0.61	0.85	0.51	0.66	0.88
	HiCseg	0.56	0.64	0.90	0.53	0.66	0.94
	Spectral	0.53	0.59	0.89	0.51	0.62	0.91
CH12LX	EmbedTAD	0.22	0.34	0.91	0.28	0.43	0.94
	ClusterTAD	0.22	0.35	0.92	0.29	0.44	0.93
	IC-Finder	0.21	0.32	0.93	0.28	0.40	0.96
	CASPIAN	0.21	0.32	0.85	0.28	0.40	0.86
	TopDom	0.21	0.33	0.94	0.29	0.42	0.95
	Armatus	0.21	0.32	0.90	0.28	0.40	0.91
	HiCseg	0.22	0.34	0.93	0.28	0.42	0.95
	Spectral	0.21	0.32	0.94	0.28	0.40	0.96

Supplementary Table 4 Running time in seconds. EmbedTAD takes minimal running time on average compared to other TAD callers.

Algorithm	GM12878 (seconds)		CH12LX (seconds)		Average (seconds)
	5Kb	10Kb	5Kb	10Kb	
EmbedTAD	298	117	529	266	302.5
ClusterTAD	5864	804	12989	1514	5292.75
IC-Finder	3719	246	1886	618	1617.25
CASPIAN	5521	678	19327	2407	6983.25
TopDom	45	12	99	24	45
Armatus	118	24	295	57	123.5
HiCseg	2918	280	7395	904	2874.25
Spectral	30	11	59	20	30

Supplementary Table 5 Memory consumption in Mbs. EmbedTAD consumes a minimal amount of memory on average compared to other TAD callers.

Algorithm	GM12878 (Mb)		CH12LX (Mb)		Average (Mb)
	5Kb	10Kb	5Kb	10Kb	
EmbedTAD	8652	2882	6674	3474	5420.5
ClusterTAD	34681	23680	34603	31605	31142.25
IC-Finder	16194	4732	20199	8746	12467.75
CASPIAN	8603	7740	6599	6598	7385
TopDom	5381	3491	10714	5440	6256.5
Armatus	6190	4035	9343	5933	6375.25
HiCseg	16766	6369	52022	16242	22849.75
Spectral	5115	3571	9678	7291	6413.75

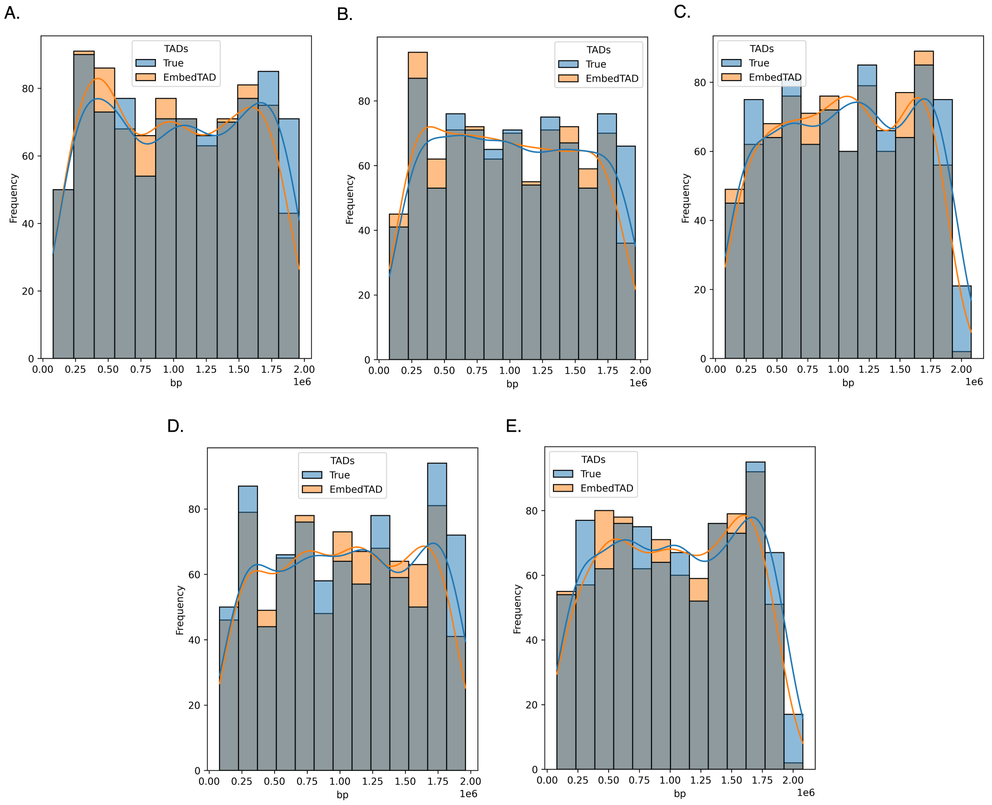

Supplementary Figure 1 TAD size distribution using In-silico Hi-C data. EmbedTAD TAD size distribution is consistent with the True TADs across 4 (A), 8 (B), 12 (C), 16 (D) and 20 (E) noise level.

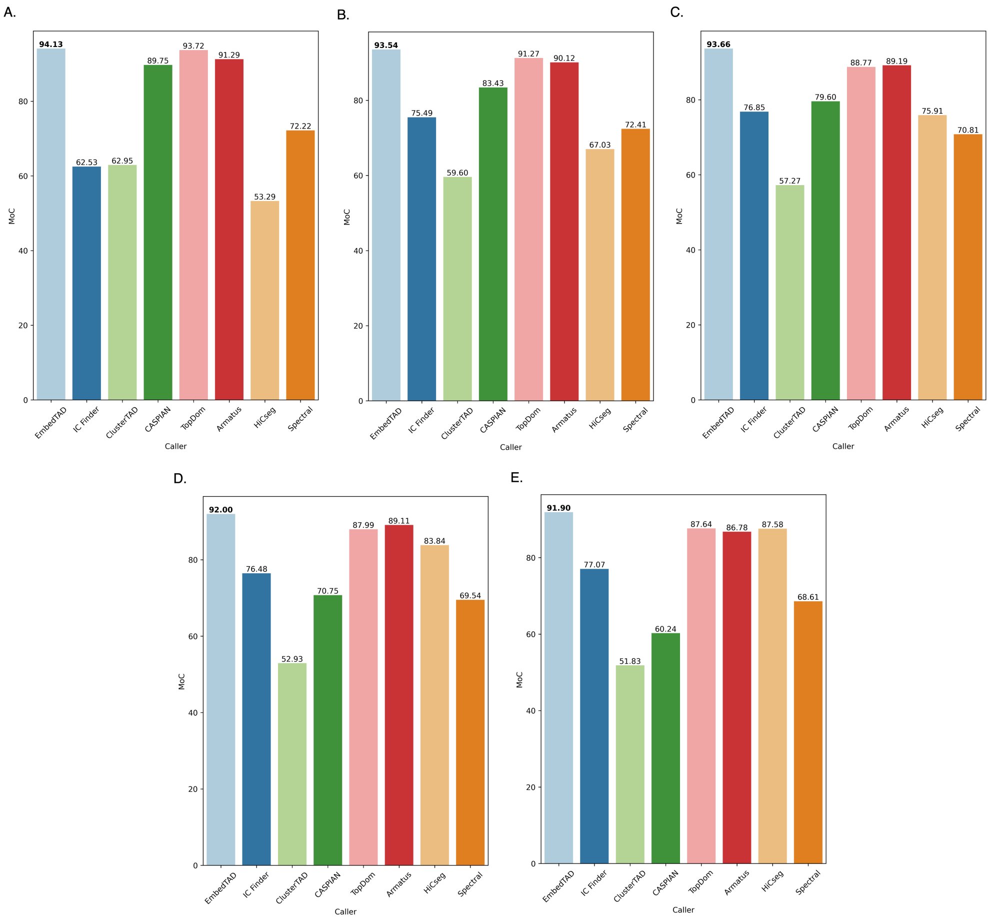

Supplementary Figure 2 MoC comparison with 7 state-of-the-arts using In-silico Hi-C data. EmbedTAD showed a higher MoC compared with the 7 state-of-the-art TAD callers across 4 (A), 8 (B), 12 (C), 16 (D) and 20 (E) noise level.

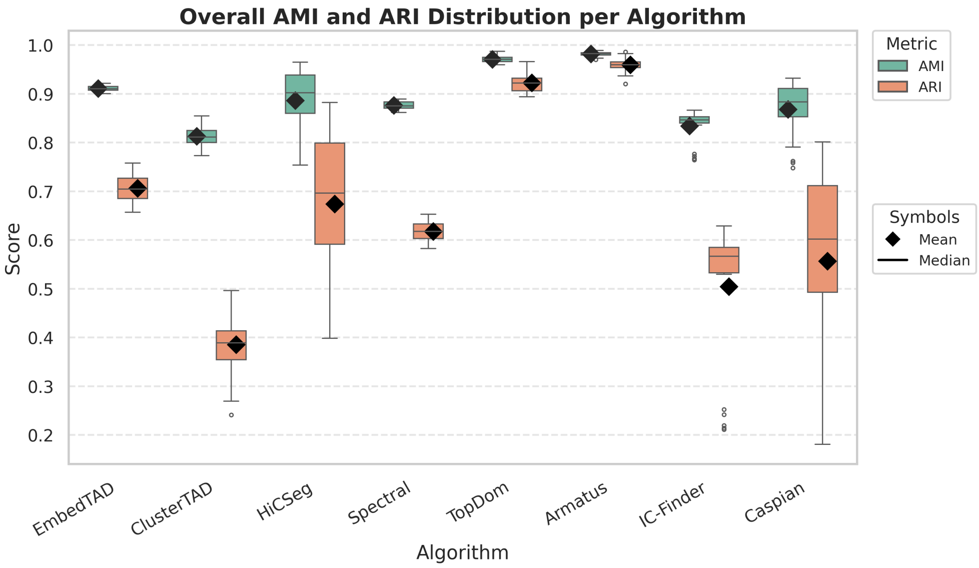

Supplementary Figure 3 AMI and ARI scores using synthetic Hi-C data. Boxplot shows the mean and median values of AMI and ARI scores, where EmbedTAD showed competitive results without any outliers and nearly identical mean and median values.

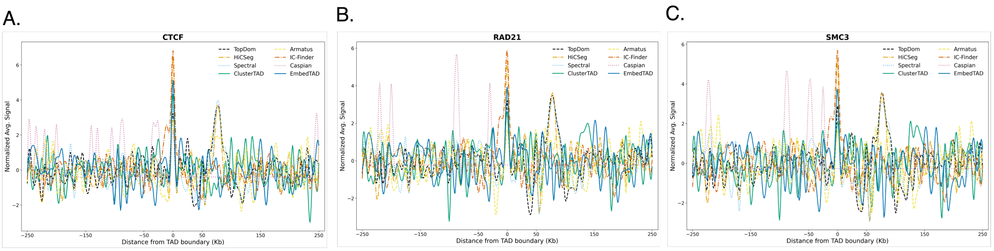

Supplementary Figure 4 ChIP signal comparison of TAD callers using GM12878 chromosome 19 at 5Kb resolution. Comparison of CTCF, RAD21 and SMC3 signal from -250Kb to +250Kb with other TAD callers where EmbedTAD shows enrichment signal at boundary.

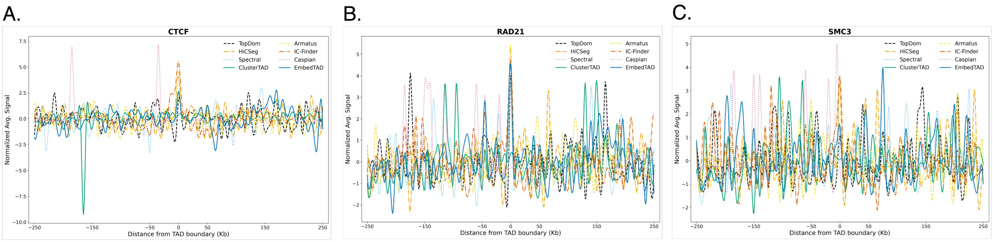

Supplementary Figure 5 ChIP signal comparison of TAD callers using CH12LX chromosome 18 at 5Kb resolution. Comparison of CTCF, RAD21 and SMC3 signal from -250Kb to +250Kb with other TAD callers where EmbedTAD shows enrichment signal at boundary.

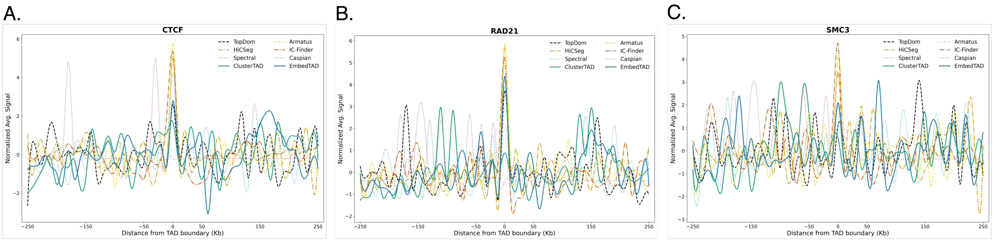

Supplementary Figure 6 ChIP signal comparison of TAD callers using CH12LX chromosome 18 at 10Kb resolution. Comparison of CTCF, RAD21 and SMC3 signal from -250Kb to +250Kb with other TAD callers where EmbedTAD shows enrichment signal at boundary.

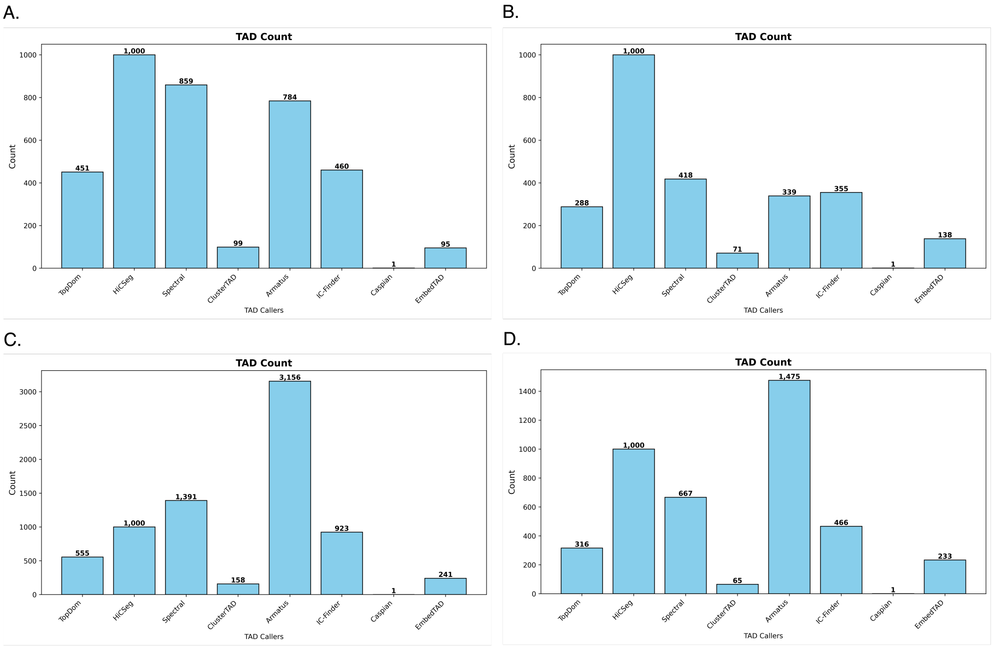

Supplementary Figure 7 Number of TADs. Bar plots show the detected TADs with different algorithms using: (A) GM12878 chromosome 19 at 5Kb, (B) GM12878 chromosome 19 at 10Kb, (C) CH12LX chromosome 18 at 5Kb, and (D) CH12LX chromosome 18 at 10Kb.

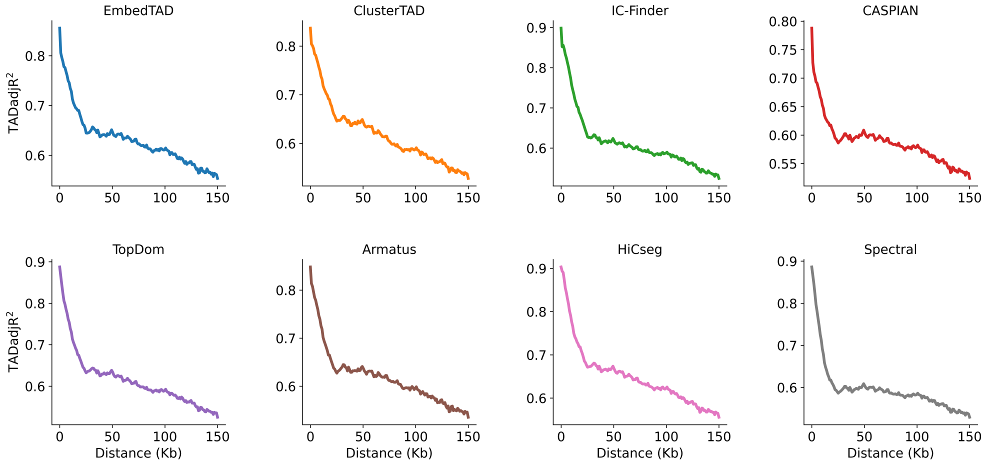

Supplementary Figure 8 $TADadjR^2$ **comparison of TAD callers using GM12878 chromosome 19 at 5Kb resolution.** Line plots show decay of adjusted R^2 score from 0 to 1.5Mb region across 8 TAD callers.

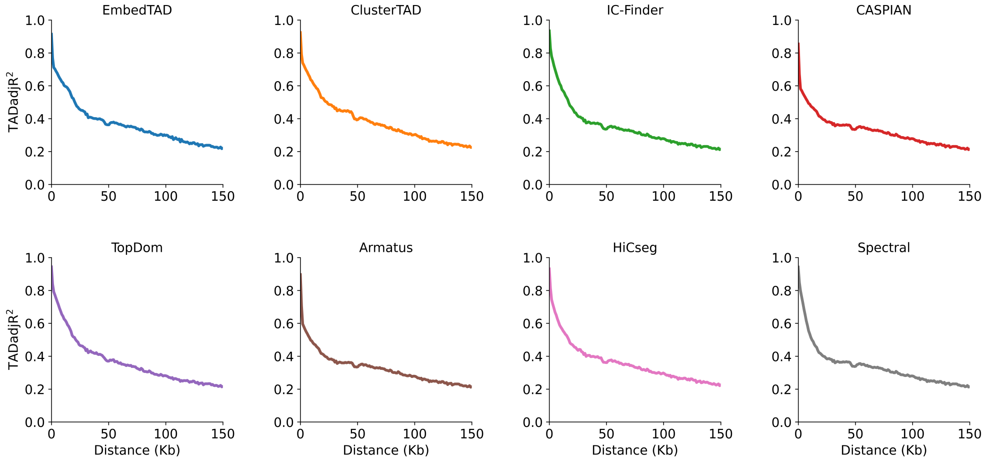

Supplementary Figure 9 $TADadjR^2$ **comparison of TAD callers using CH12LX chromosome 18 at 5Kb resolution.** Line plots show decay of adjusted R^2 score from 0 to 1.5Mb region across 8 TAD callers.

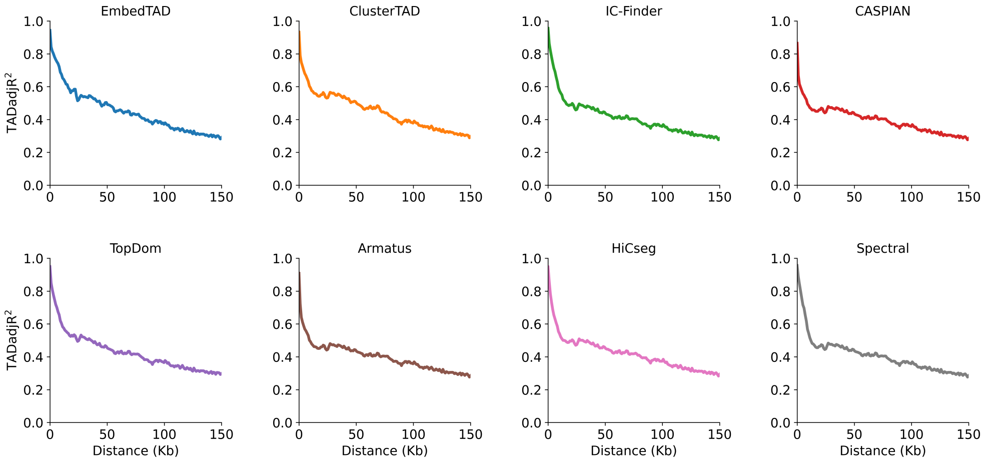

Supplementary Figure 10 $TADadjR^2$ **comparison of TAD callers using CH12LX chromosome 18 at 10Kb resolution.** Line plots show decay of adjusted R^2 score from 0 to 1.5Mb region across 8 TAD callers.

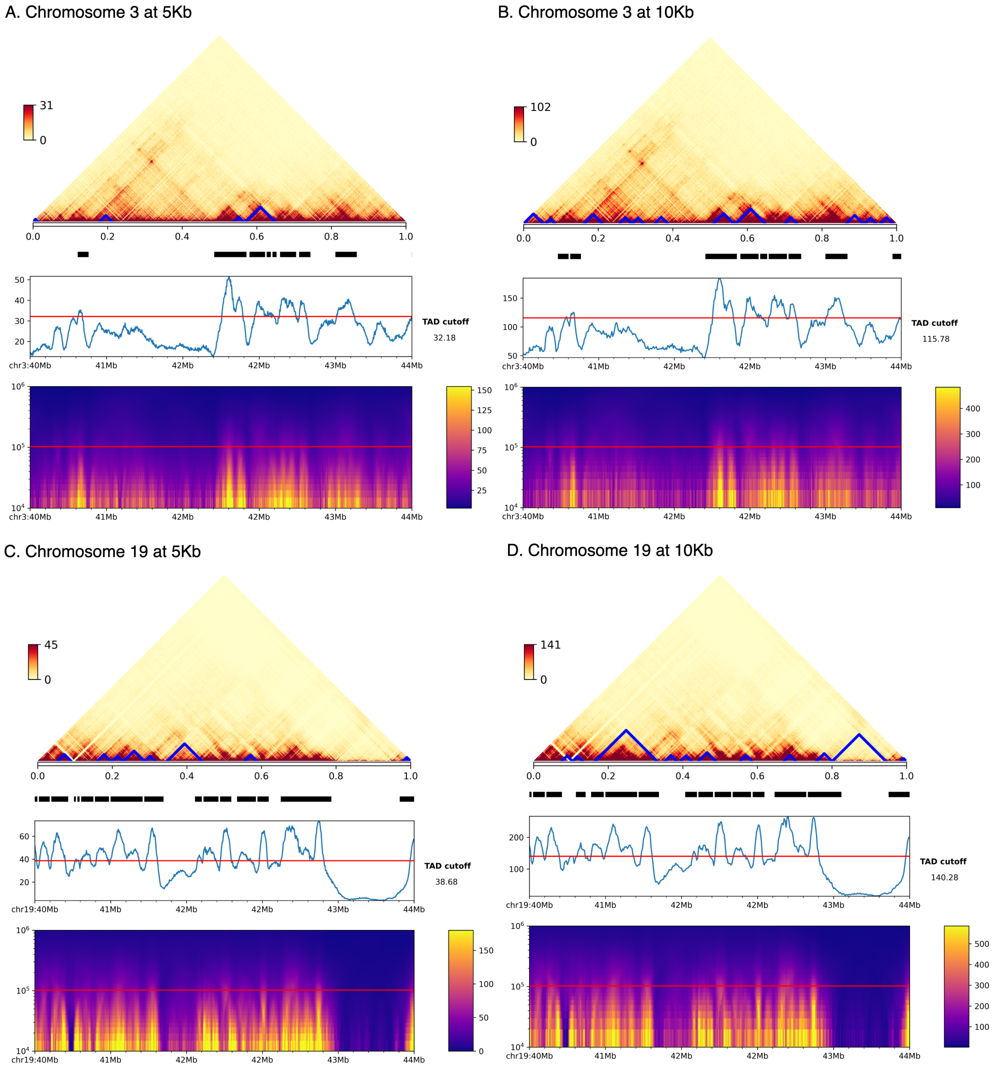

Supplementary Figure 11 TADs agreement with insulation score of EmbedTAD on GM12878 chromosome 3, 19 at 5Kb and 10Kb resolutions. Insulation Score (IS) on GM12878 from 40Mb to 44Mb region.EmbedTAD's detected TADs are annotated with blue lines (top), line plot shows IS over the region with TAD cutoff threshold, and bars indicate currently called TADs (middle), and heatmap with IS score (bottom). Within this region, EmbedTAD showed significant agreement between detected TADs and IS scores.

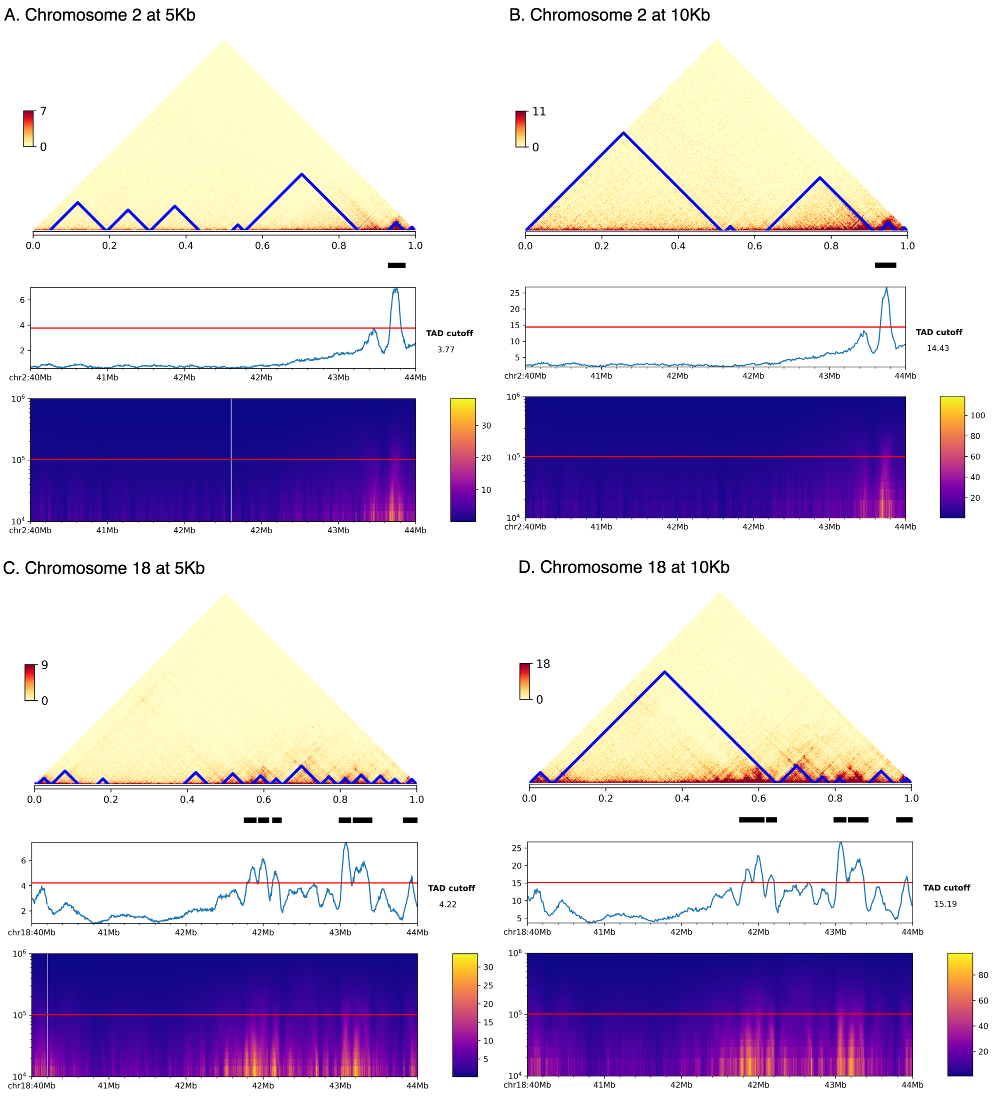

Supplementary Figure 12 TADs agreement with insulation score of EmbedTAD on CH12LX chromosome 2, 18 at 5Kb and 10Kb resolution. Insulation Score (IS) on CH12LX from 40Mb to 44Mb region. EmbedTAD's detected TADs are annotated with blue lines (top), line plot shows IS over the region with TAD cutoff threshold, and bars indicate currently called TADs (middle), and heatmap with IS score (bottom). Within this region, EmbedTAD showed significant agreement between detected TADs and IS scores.

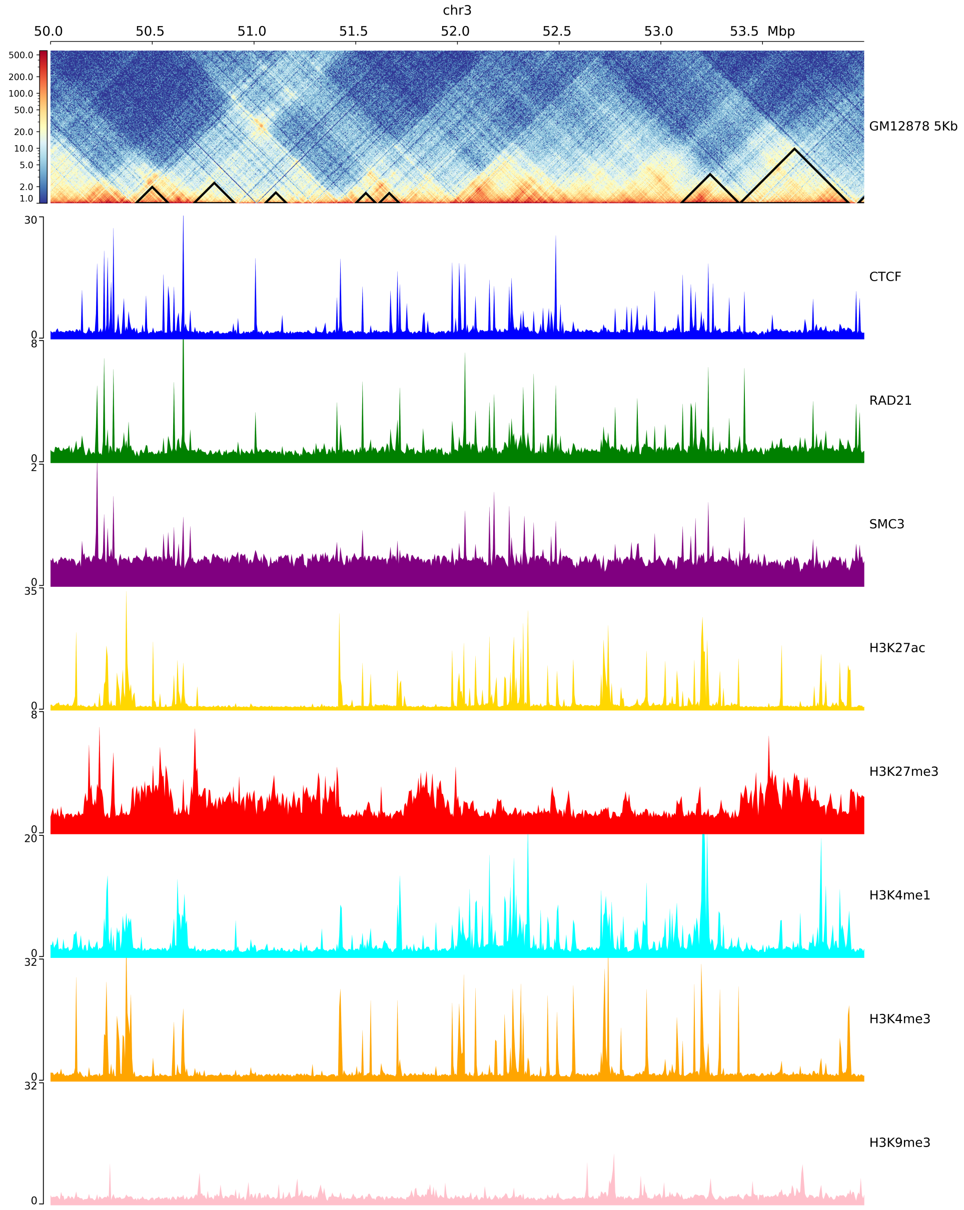

Supplementary Figure 13 Biological validation of EmbedTAD using different ChIP signal. Visualization of EmbedTAD identified TADs on GM12878 chromosome 3 at 5Kb resolution with different ChIP signal from 50Mb to 54Mb region.

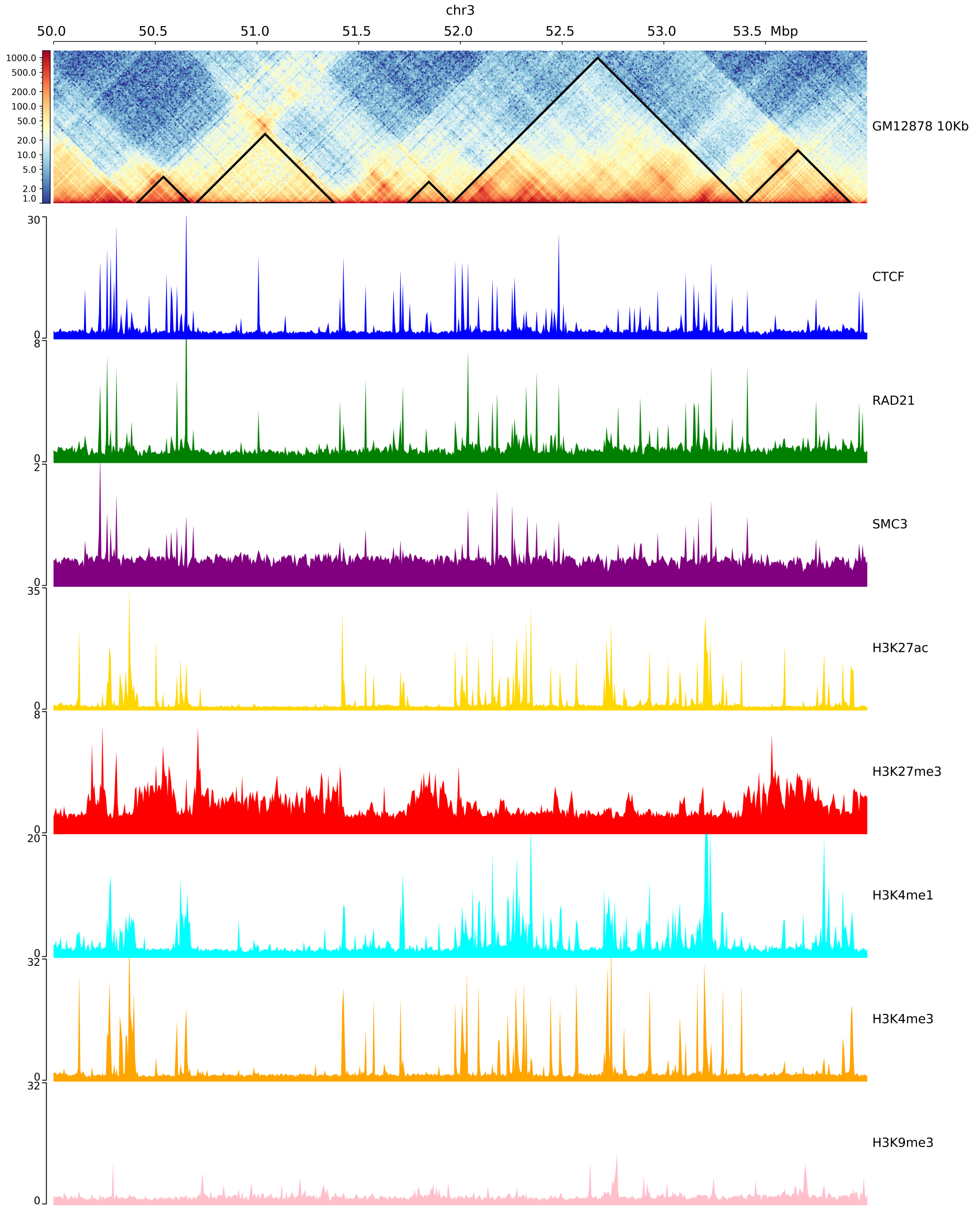

Supplementary Figure 14 Biological validation of EmbedTAD using different ChIP signal. Visualization of EmbedTAD identified TADs on GM12878 chromosome 3 at 10Kb resolution with different ChIP signal from 50Mb to 54Mb region.

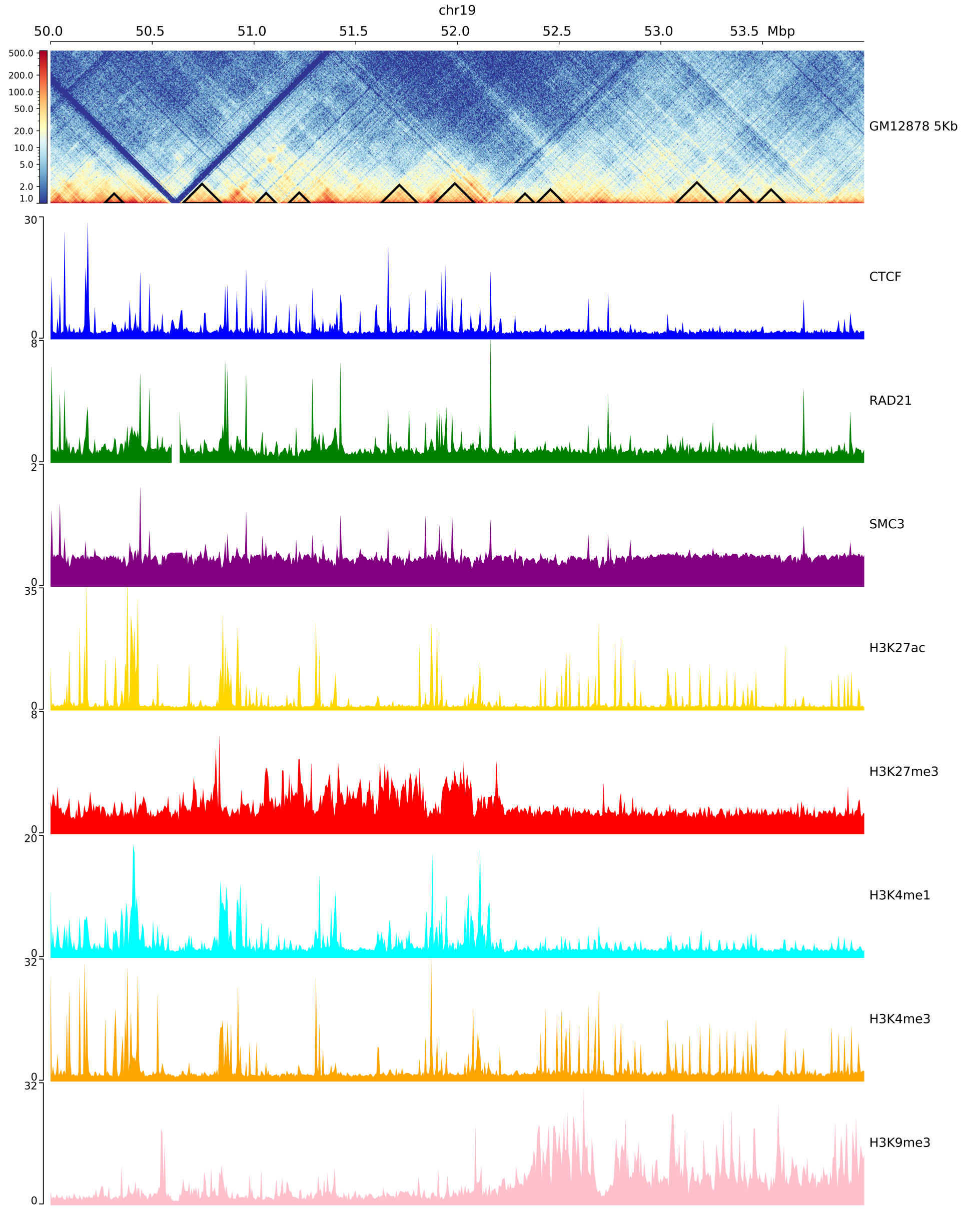

Supplementary Figure 15 Biological validation of EmbedTAD using different ChIP signal. Visualization of EmbedTAD identified TADs on GM12878 chromosome 19 at 5Kb resolution with different ChIP signal from 50Mb to 54Mb region.

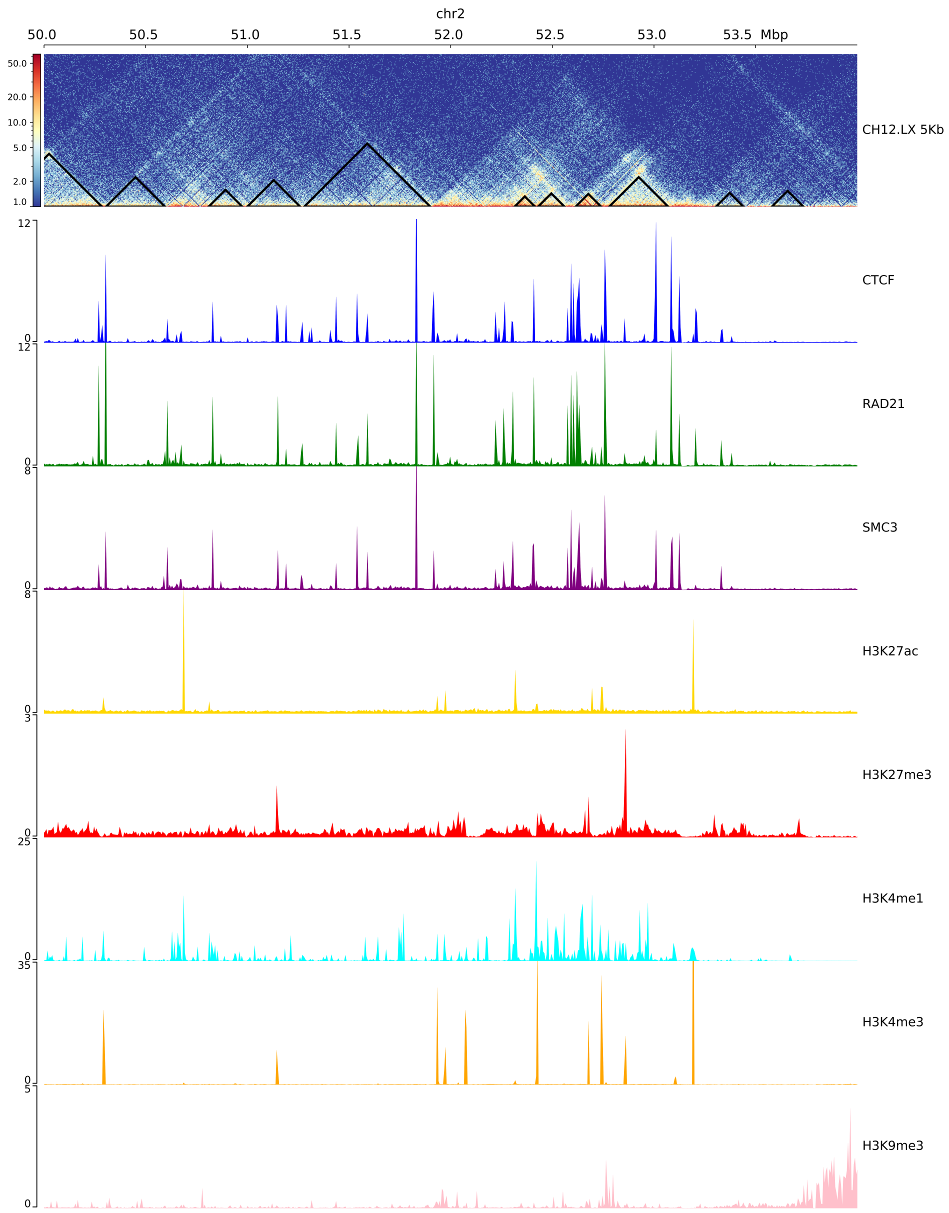

Supplementary Figure 16 Biological validation of EmbedTAD using different ChIP signal. Visualization of EmbedTAD identified TADs on CH12LX chromosome 2 at 5Kb resolution with different ChIP signal from 50Mb to 54Mb region.

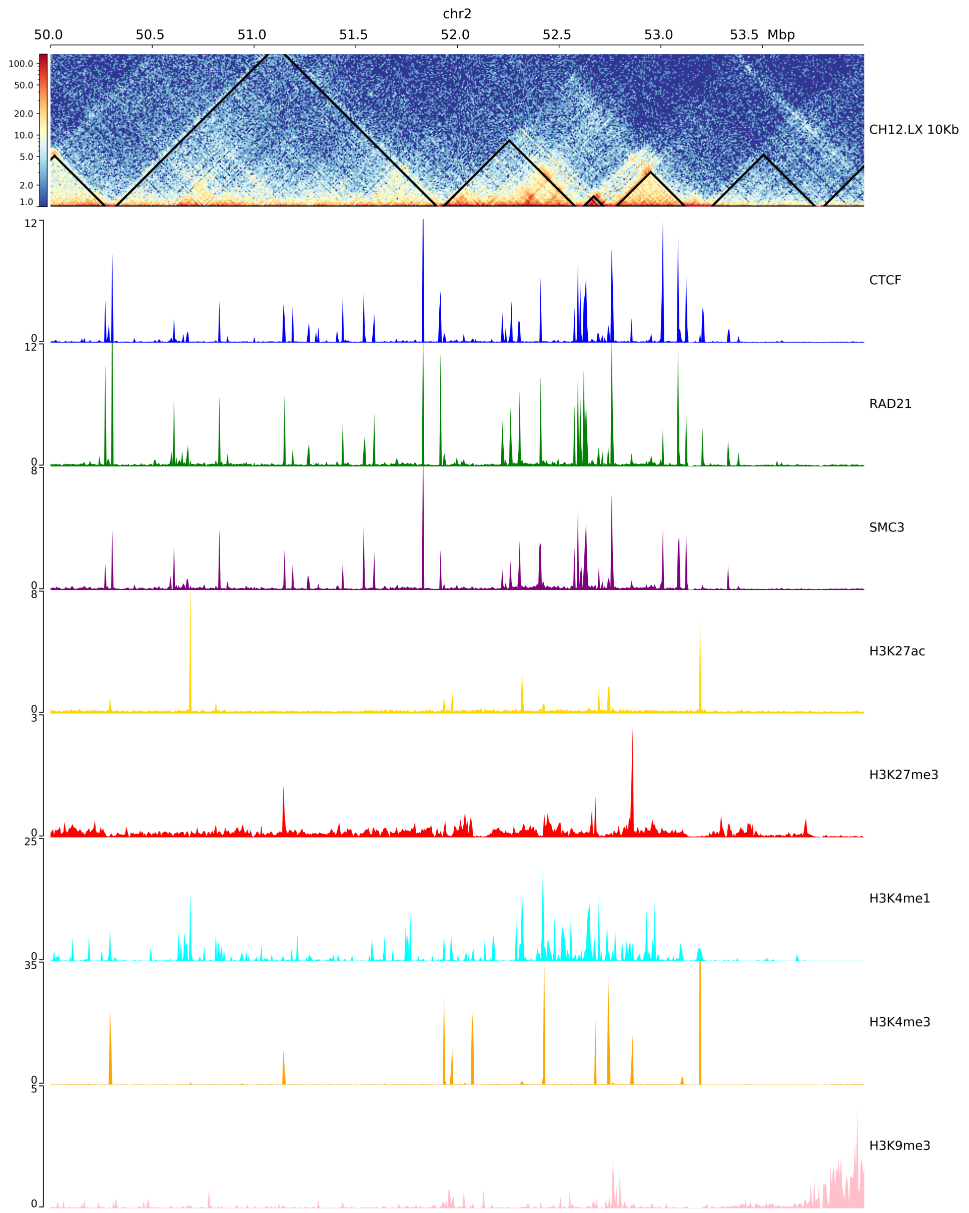

Supplementary Figure 17 Biological validation of EmbedTAD using different ChIP signal. Visualization of EmbedTAD identified TADs on CH12LX chromosome 2 at 10Kb resolution with different ChIP signal from 50Mb to 54Mb region.

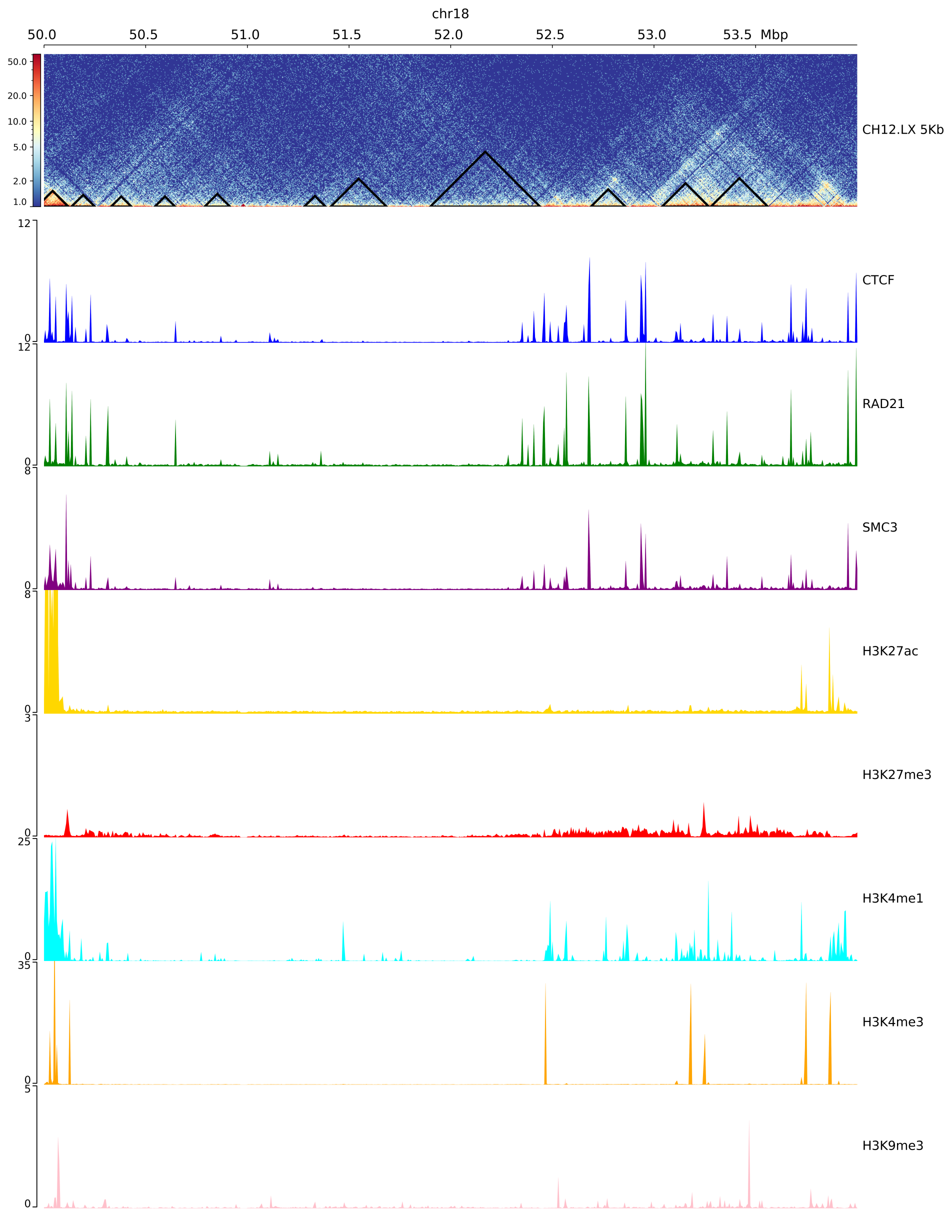

Supplementary Figure 18 Biological validation of EmbedTAD using different ChIP signal data. Visualization of EmbedTAD identified TADs on CH12LX chromosome 18 at 5Kb resolution with different ChIP signal from 50Mb to 54Mb region.

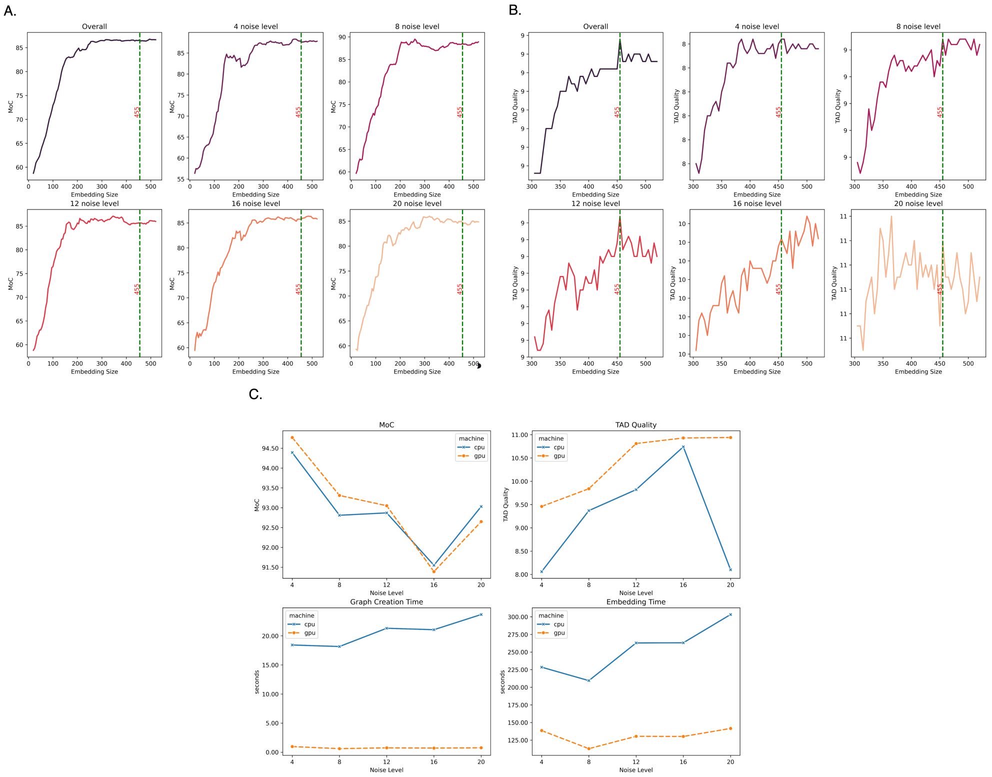

Supplementary Figure 19 Hyperparameter search using In-silico Hi-C data at varying noise level (4, 8, 12, 16, and 20). A. MoC analysis at different noise levels at different embedding sizes (20 to 520). Our analysis shows optimum results at 455 embedding sizes across all noise levels. **B**. TAD Quality analysis at different noise levels at different embedding sizes (300 to 520). Our analysis shows optimum results at 455 embedding sizes across all noise levels. **C**. Computational analysis of CPU vs GPU implementation where GPU performs faster than CPU maintaining almost the same MoC and TAD quality score.

References

[1] Forcato, M., Nicoletti, C., Pal, K., Livi, C.M., Ferrari, F., Bicciato, S.: Comparison of computational methods for hi-c data analysis. Nature methods **14**(7), 679–685 (2017)

[2] Pfitzner, D., Leibbrandt, R., Powers, D.: Characterization and evaluation of similarity measures for pairs of clusterings. Knowledge and Information Systems **19**, 361–394 (2009)

[3] Oluwadare, O., Cheng, J.: Clustertad: an unsupervised machine learning approach to detecting topologically associated domains of chromosomes from hi-c data. BMC bioinformatics **18**, 1–14 (2017)

[4] Dixon, J.R., Selvaraj, S., Yue, F., Kim, A., Li, Y., Shen, Y., Hu, M., Liu, J.S., Ren, B.: Topological domains in mammalian genomes identified by analysis of chromatin interactions. Nature **485**(7398), 376–380 (2012)

[5] Liu, E., Lyu, H., Liu, Y., Fu, L., Cheng, X., Yin, X.: Identifying tad-like domains on single-cell hi-c data by graph embedding and changepoint detection. Bioinformatics **40**(3), 138 (2024)

[6] Li, X., Zeng, G., Li, A., Zhang, Z.: Detoki identifies and characterizes the dynamics of chromatin tad-like domains in a single cell. Genome biology **22**(1), 217 (2021)
