# Supplementary material for: EmbedTAD Using Graph Embedding and Unsupervised Learning to Identify TADs from High-Resolution Hi-C Data
Source: Commun Biol. 2025 Dec 9;9:7. doi: 10.1038/s42003-025-09224-z (PMC12764586; doi:10.1038/s42003-025-09224-z)
Supplement: Supplementary file 3 — Description of Additional Supplementary Files [file 42003_2025_9224_MOESM3_ESM.pdf]

Description of Additional Supplementary Files

File name: Supplementary Data 1

Description: Initial parameter search based on Measure of Concordance (MoC) metric from 20 to 520 embedding size using in-silico Hi-C dataset with 5 different noise level.

File name: Supplementary Data 2

Description: Parameter search based on Measure of Concordance (MoC) and TAD Quality (TQ) from 305 to 520 embedding size using in-silico Hi-C dataset with 5 different noise level.

File name: Supplementary Data 3

Description: Determining optimal embedding size based on Measure of Concordance (MoC) and TAD Quality (TQ) results using in-silico Hi-C dataset.
